# Role of Stem Cells in Human Uterine Leiomyoma Growth

**DOI:** 10.1371/journal.pone.0036935

**Published:** 2012-05-03

**Authors:** Masanori Ono, Wenan Qiang, Vanida Ann Serna, Ping Yin, John S. Coon, Antonia Navarro, Diana Monsivais, Toshiyuki Kakinuma, Matthew Dyson, Stacy Druschitz, Kenji Unno, Takeshi Kurita, Serdar E. Bulun

**Affiliations:** Division of Reproductive Biology Research, Department of Obstetrics and Gynecology, Feinberg School of Medicine at Northwestern University, Chicago, Illinois, United States of America; University of Genoa Medical School - San Martino Hospital, Italy

## Abstract

**Background:**

Uterine leiomyoma is the most common benign tumor in reproductive-age women. Each leiomyoma is thought to be a benign monoclonal tumor arising from a single transformed myometrial smooth muscle cell; however, it is not known what leiomyoma cell type is responsible for tumor growth. Thus, we tested the hypothesis that a distinct stem/reservoir cell-enriched population, designated as the leiomyoma-derived side population (LMSP), is responsible for cell proliferation and tumor growth.

**Principal Findings:**

LMSP comprised approximately 1% of all leiomyoma and 2% of all myometrium-derived cells. All LMSP and leiomyoma-derived main population (LMMP) but none of the side or main population cells isolated from adjacent myometrium carried a mediator complex subunit 12 mutation, a genetic marker of neoplastic transformation. Messenger RNA levels for estrogen receptor-α, progesterone receptor and smooth muscle cell markers were barely detectable and significantly lower in the LMSP compared with the LMMP. LMSP alone did not attach or survive in monolayer culture in the presence or absence of estradiol and progestin, whereas LMMP readily grew under these conditions. LMSP did attach and survive when directly mixed with unsorted myometrial cells in monolayer culture. After resorting and reculturing, LMSP gained full potential of proliferation. Intriguingly, xenografts comprised of LMSP and unsorted myometrial smooth muscle cells grew into relatively large tumors (3.67±1.07 mm^3^), whereas xenografts comprised of LMMP and unsorted myometrial smooth muscle cells produced smaller tumors (0.54±0.20 mm^3^, p<0.05, n = 10 paired patient samples). LMSP xenografts displayed significantly higher proliferative activity compared with LMMP xenografts (p<0.05).

**Conclusions:**

Our data suggest that LMSP, which have stem/reservoir cell characteristics, are necessary for *in vivo* growth of leiomyoma xenograft tumors. Lower estrogen and progesterone receptor levels in LMSP suggests an indirect paracrine effect of steroid hormones on stem cells via the mature neighboring cells.

## Introduction

Uterine leiomyomas, the most common pelvic tumor in women, are benign smooth muscle tumors originating from the myometrium [1]. Uterine leiomyomas occur in 60% of women by the age of 45 years and cause symptoms in approximately 30% of the cases [2], [3]. These symptoms include pelvic pain, discomfort, and abnormal bleeding. Uterine leiomyomas are also an important cause of infertility and they are the most common medical reason for hysterectomy [3], [4]. Despite the high prevalence, the exact pathophysiology of uterine leiomyomas is still unknown.

Somatic stem cells are a subset of cells residing in normal adult tissues that, through asymmetric division, retain their ability to self-renew while producing daughter cells that go on to differentiate and play a role in tissue regeneration and repair [5], [6]. Likewise, tumor initiating cells are a subset of cells within a tumor cell population, which, also through asymmetric division, retain the ability to reconstitute tumors [7], [8]. The side population (SP) phenotype was first described in bone marrow, where a somatic stem cell population was identified based on its ability to extrude the DNA binding dye Hoechst 33342, a phenomenon that is associated with the expression of ATP-binding cassette transporter G2 [8]. In general, the SP phenotype is thought to be a universal marker of somatic stem cells and has been used to isolate them from many adult tissues, such as the myometrium, endometrium and mammary gland [9], [10], [11], [12].

Leiomyomas are thought to be monoclonal tumors arising from the myometrium; however, it is not known what cell population in the myometrium gives rise to these tumors [13], [14]. Several recurrent genetic aberrations, such as trisomy of chromosome 12, deletions in 7q, and mutation affecting the mediator complex subunit 12 (*MED12*) or the high mobility group AT-hook 2 (*HMGA2*) gene were reported in uterine leiomyomas [15], [16], [17], [18]. As in other diseases, these genetic abnormalities and tumor stem cells are considered to play pivotal roles in the tumorigenesis of leiomyoma. To investigate the possible role of stem cells in human uterine leiomyoma growth, we examined leiomyoma derived SP cells (LMSP).

## Methods

### Preparation of human myometrial and leiomyoma cells

Myometrium and leiomyoma were obtained at surgery from women (range 27–52 years) undergoing hysterectomy or myomectomy, in addition to some basic endocrine information (e.g., day of menstrual cycle, parity, whether oral contraception is being taken). Written informed consent was obtained from each patient and the use of human tissue specimens was approved by the Institutional Review Board for Human Research at Northwestern University, Chicago, IL. None of these cases had any previous history of uterine cancer, and all samples were confirmed by histopathological examination to be free of malignancy. The tissues were prepared as previously described [9].

**Table 1:** List of antibodies used in this study.

Antgigen	Clone	Isotype	Supplier
CD31 (FACS)	WM59	FITC-conjugated mouse IgG1	BD Pharmingen (San Jose, CA)
CD45 (FACS)	HI30	FITC-conjugated mouse IgG1	BD Pharmingen
CD73 (FACS)	AD2	PE-conjugated mouse IgG1	BD Pharmingen
CD90 (FACS)	5.00E+10	PE-conjugated mouse IgG1	BD Pharmingen
CD105 (FACS)	266	FITC-conjugated mouse IgG1	BD Pharmingen
STRO-1 (FACS)	STRO-1	mouse IgM	R & D Systems (Minneapolis, MN)
α-smooth muscle actin	1A4	mouse IgG2a	DAKO Cytomation (Glostrup, Denmark)
Estrogen recetor 1	SP1	rabbit IgG	Lab Vision (Kalamazoo, MI)
Progesterone receptor	SP2	rabbit IgG	Lab Vision

**Table 2:** List of primer sets used in this study.

Gene	Primer sets	Accession number
Estrogen receptor 1 *(ESR1)*	5′-CACCAACCAGTGCACCATCATTG-3′	NM_001122740
	5′-AAGGTTGGCAGCTCTCATGTC-3′	
Estrogen receptor 2 *(ESR2)*	5′-CCATGATCCTGCTCAATTCC-3′	NM_001437
	5′-CTCTTGGCAATCACCCAAAC-3′	
Progesterone receptor *(PGR)*	5′-CATTTGCACAAACCTGATGG-3′	NM_001202474
	5′-CATGGTGTACAAGGCCACTG-3′	
α smooth muscle actin	5′-CAAGTGATCACCATCGGAAATG-3′	NM_001141945
	5′-GACTCCATCCCGATGAAGGA-3′	
Calponin	5′-TGAAGCCCCACGACATTTTT-3′	NM_001299
	5′-GGGTGGACTGCACCTGTGTA-3′	
SM22α	5′-CAAGCTGGTGAACAGCCTGTAC-3′	NM_003186
	5′-GACCATGGAGGGTGGGTTCT-3′	
Glyceraldehyde-3-phosphate dehydrogenase (*GAPDH*)	5′-GAAGGTGAAGGTCGGAGTC-3′	NM_002046
	5′-GAAGATGGTGATGGGATTTC-3′	

**Figure 1:**
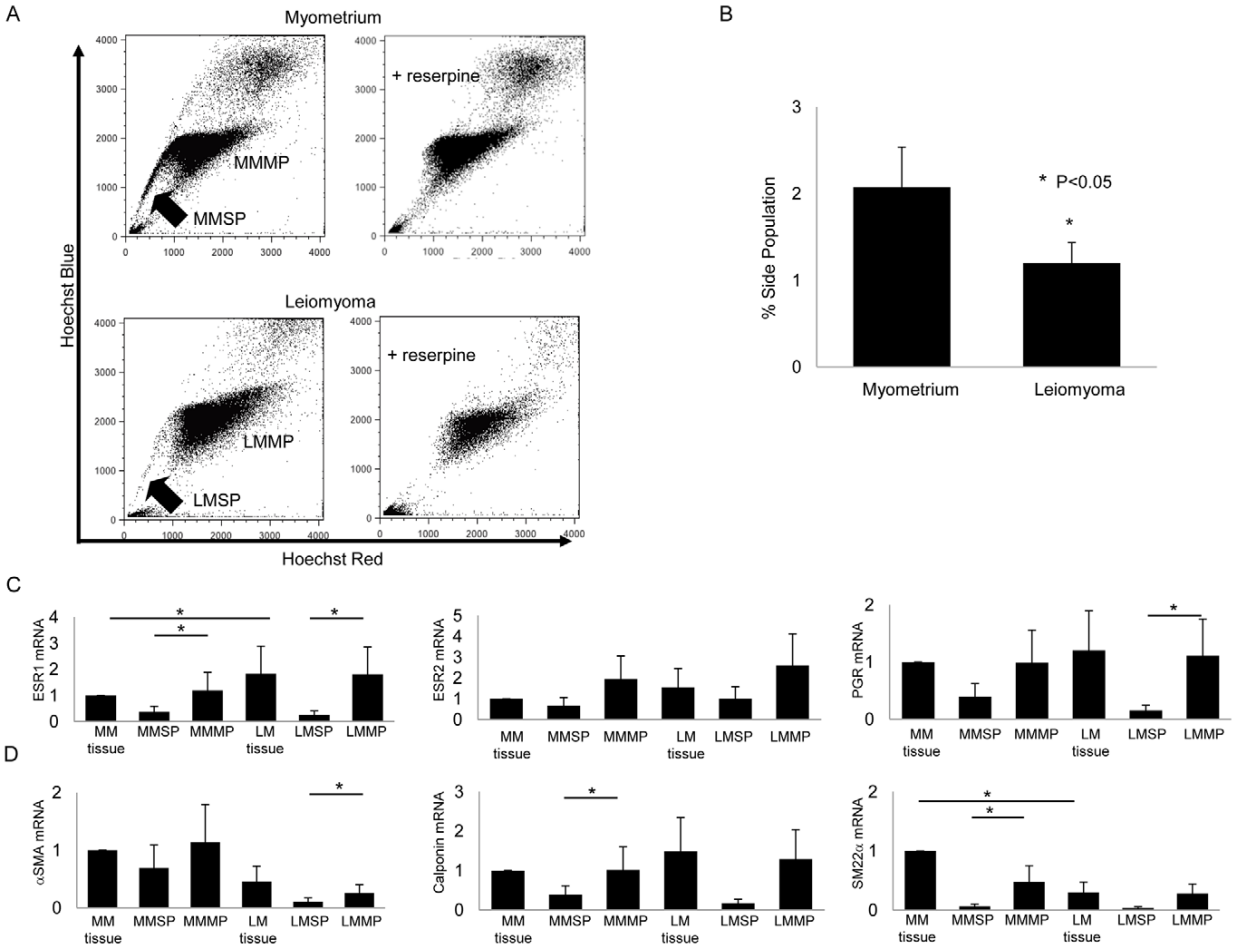
Isolation and characterization of human MMSP and LMSP. (A) (Left upper) Distribution of the SP and MP cells within all Hoechst 33342-stained living cells isolated from human myometrium. (Right upper) Addition of 50 µM reserpine resulted in the disappearance of the MMSP fraction. (Left lower) Distribution of SP and MP cells isolated from human leiomyoma. (Right lower) Addition of 50 µM reserpine resulted in the disappearance of the LMSP fraction. (B) Average % SP of normal myometrium and leiomyomas from 10 different patients are shown (2.07%±0.46 vs. 1.19%±0.23, *P*<0.05). Error bars represent SEM. (C) mRNA expression of ovarian steroid receptors and (D) smooth muscle cell markers was examined by real-time RT-PCR and normalized for GAPDH expression. Each bar indicates the mean ± SEM of the relative expression obtained from three independent experiments using three individual samples. *, *P*<0.05.

**Figure 2:**
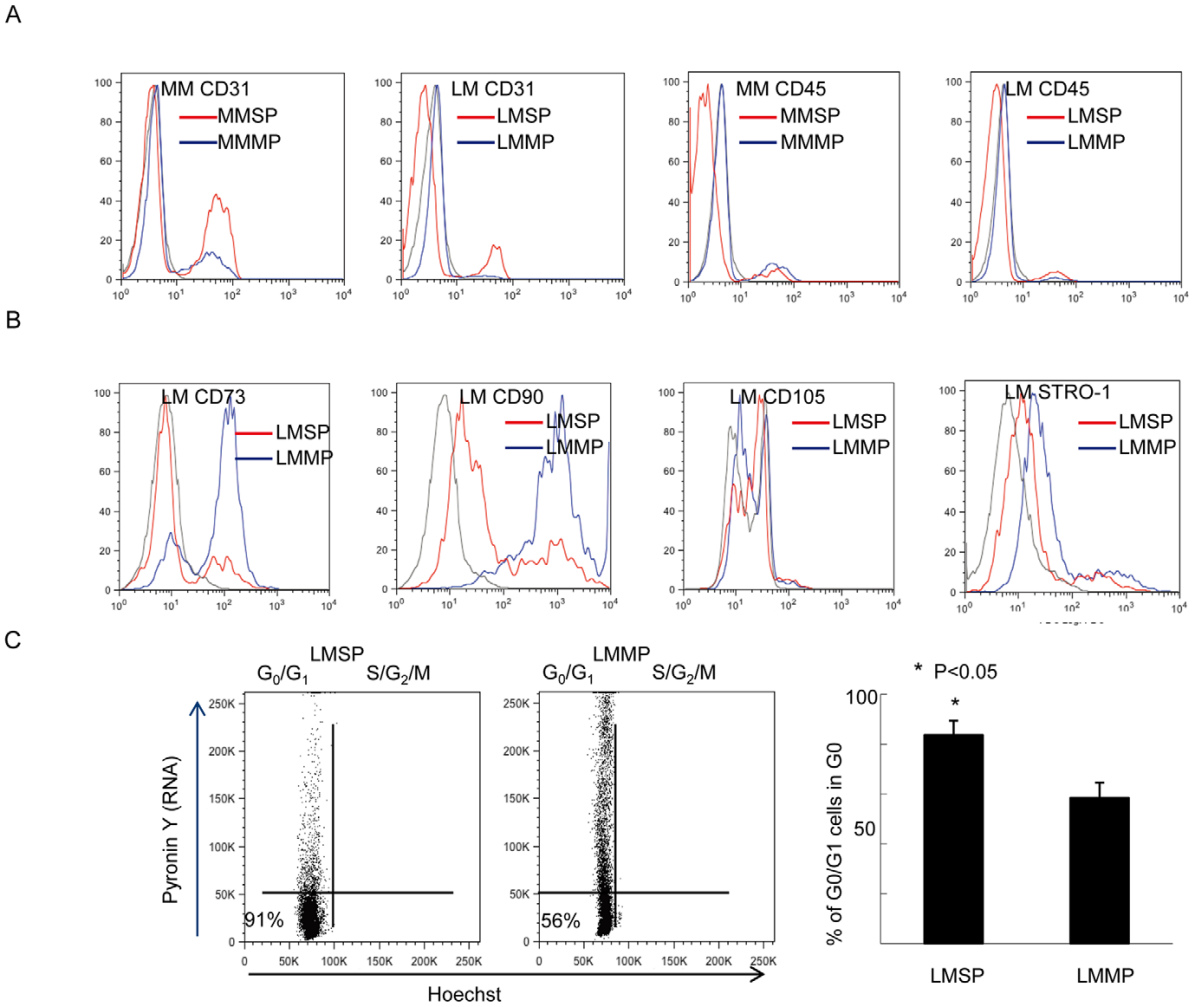
Cell surface marker antigens in LMSP. (A) Expression patterns of endothelial cell surface markers (CD31, Platelet Endothelial Cell Adhesion Molecule-1) and hematopoietic (CD45, Leukocyte common antigen) in MMSP, LMSP (red); and MMMP, LMMP (blue). In LMSP, most of the cells were negative for endothelial and hematopoietic cell markers. (B) Expression patterns of bone marrow mesenchymal stem cell surface markers (CD73, CD90, CD105, STRO-1) in MMSP, LMSP (red); and MMMP, LMMP (blue). These bone mesenchymal cell surface antigens were not able to identify LMSP. Mouse FITC-labeled IgG1 (BD Biosciences) was used as an isotypic control for staining of total myometrial or leiomyoma cells (black). (C) Cell cycle status of LMSP and LMMP was determined by Hoechst 33342 and Pyronin Y staining. The left lower quadrant corresponds to the G_0_ phase. Flow cytometry analysis revealed that 83.67%±5.73 of LMSP but only 58.50%±6.06 of LMMP were in the G_0_ phase.

**Figure 3:**
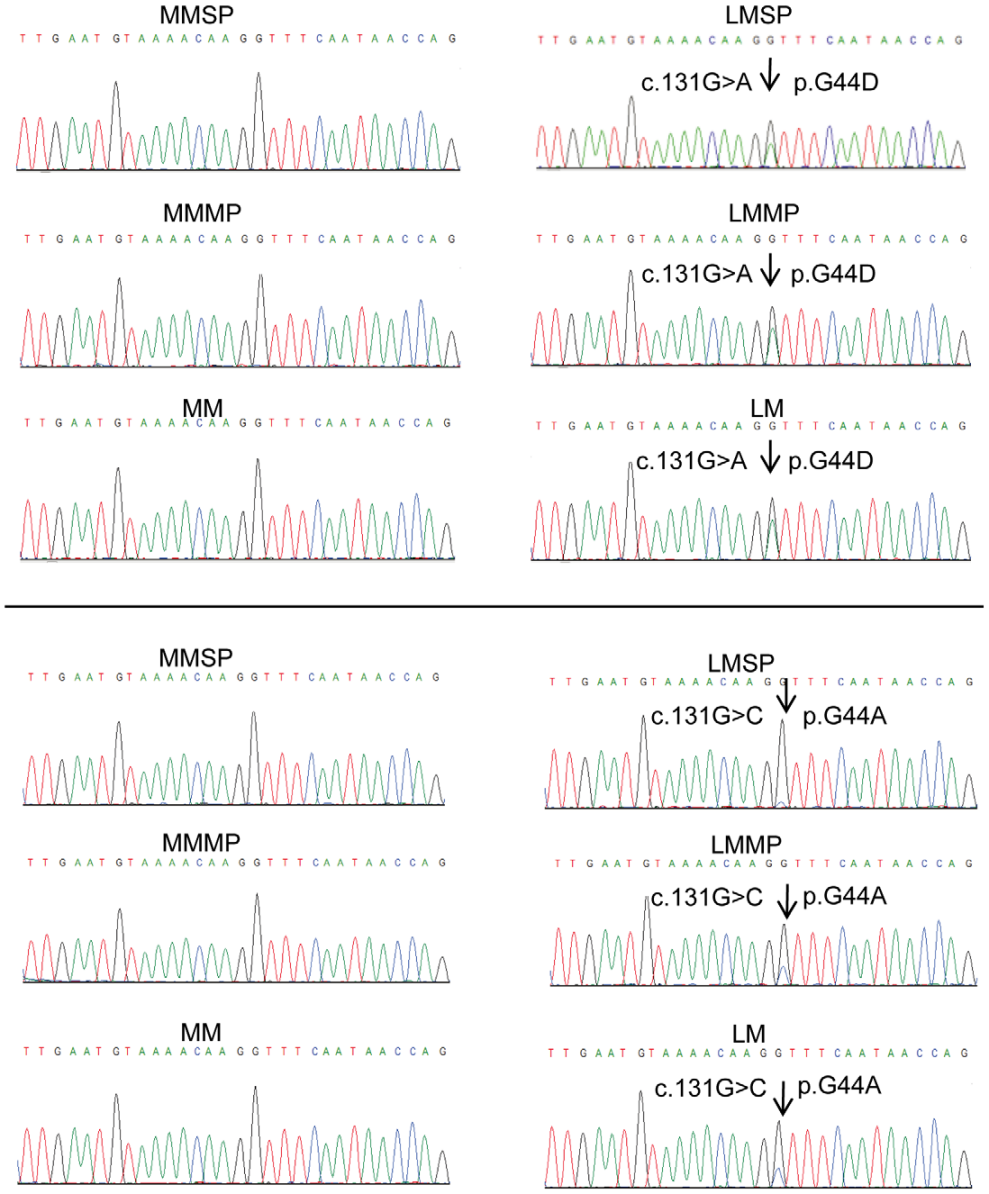
Sequence chromatograms showing somatic mutations in *MED12* codon 44 in LMSP. Examples of genomic DNA sequencing traces in codon 44-mutated samples are shown. Mutated bases are indicated by arrows.

**Figure 4:**
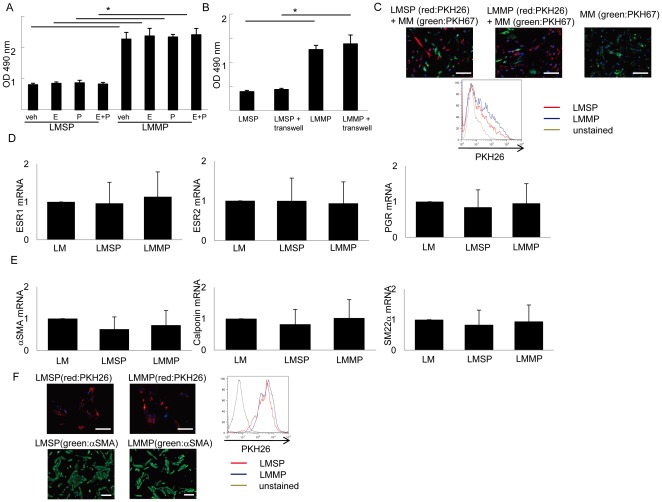
Cell culture of LMSP. (A) Effects of E_2_ and/or P on the viability of LMSP as determined by the MTS assay. Each bar indicates the mean ± SEM of the absorbance at 490 nm obtained from three independent experiments using three individual samples. *, *P*<0.05. (B) Effects of indirect co-culturing with MM on the viability of LMSP. *, *P*<0.05. (C) Side or main populations in mixed co-cultures with MM at a 1∶1 ratio were identified after initially dye-labeling each cell type. Cells originating from LMSP, LMMP, and MM in mixed co-cultures were identified using long-lasting status PKH-26 (red) or PKH-67 (green). Scale bars, 100 µm. (D) mRNA expression of the ovarian steroid receptors and (E) smooth muscle cell markers was examined by real-time RT-PCR and normalized for GAPDH expression. Each bar indicates the mean ± SEM of the relative expression obtained from three independent experiments using three individual samples. *, *P*<0.05. (F) Expression of αSMA protein in LMSP and LMMP after culturing. Scale bars, 50 µm (upper), 100 µm (lower).

**Figure 5:**
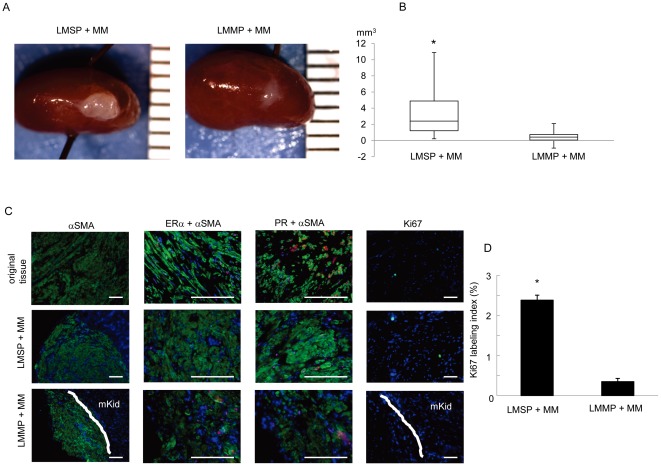
Generation of leiomyoma tumors from LMSP. (A) Macroscopic visualization of the transplanted site 12 weeks after xenotransplantation. (B) Xenografts were analyzed in terms of tumor volume. Each bar indicates the mean ± SEM. *, *P*<0.05, n = 10 paired patient samples. (C) Original tissue and generated tumor were analyzed by immunohistochemistry. Nuclei were stained with DAPI (blue). Scale bars, 100 µm. D, Ki 67 labeling index was calculated in transplanted tumors.

### Hoechst 33342 and Pyronin Y (PY) staining

The dissociated cells were re-suspended at a concentration of 2×10^6^ cells/ml in calcium- and magnesium-free Hanks-balanced salt solution containing 2% fetal bovine serum (FBS). Hoechst 33342 (Sigma-Aldrich, St. Louis, MO) was then added at a final concentration of 5 µg/ml and the sample was incubated at 37°C for 90 min. A parallel aliquot was stained with Hoechst 33342 dye in the presence of 50 µM reserpine (Sigma-Aldrich). After incubation, the cells were centrifuged at 1500 rpm for 7 minutes, re-suspended in 2 ml of cold FACS solution, and further incubated with 1 µg/ml propidium iodide (PI, Sigma-Aldrich) to label non-viable cells. The cells were kept on ice at all times after staining with the Hoechst 33342 dye. The Hoechst dye- and PI-treated cells were subjected to flow cytometric analysis to separate the side population of myometrial cells (MMSP), main population of myometrial cells (MMMP), LMSP, and main population of leiomyoma cells (LMMP). For co-staining with Hoechst 33342 and PY, sorted LMSP and LMMP were washed twice in FACS solution, incubated with 1 µg/mL Hoechst 33342 together with 50 µM reserpine at 37°C for 45 minutes, and then, without any additional washing, incubated with 3.3 µM PY (Polysciences, Warrington, PA) for another 45 minutes. The cells were washed once in an excess volume of FACS solution and subjected to FACS analysis by MoFlo (Cytomation, Fort Collins, CO) and LSR Fortessa (BD Biosciences, San Jose, CA). Cells co-treated with Hoechst and reserpine or treated with PY alone were used as negative controls.

### FACS analysis

Myometrial and leiomyoma cells were sorted by MoFlo and analyzed using the Flowjo software (Tree Star Ashland, OR). Myometrial and leiomyoma cells were re-suspended in FACS solution at 1–5×10^7^ cells/ml. The antibodies used for FACS were conjugated with fluorescein isothiocyanate (FITC) or phycoerythrin (PE) (**Table 1**). Hoechst 33342 was excited at 350 nm, and the fluorescence emission was detected using 405/BP (band pass) 30 and 570/BP20 optical filters for Hoechst blue and Hoechst red, respectively. A 550-nm long-pass dichroic mirror (Omega Optical Inc., Brattleboro, VT) was used to separate the emission wavelengths. Both Hoechst blue and red fluorescence intensities are shown on a linear scale. PI fluorescence was measured through a 630/BP30 optical filter after excitation at 488 nm with an argon laser, and a live cell gate was defined to exclude PI-positive cells. After collecting 1×10^5^ events, the SP population was defined as previously reported [11], [19], [20]. Forward scatter, side scatter, and PI gating excluded residual erythrocytes, debris, doublets, and dead cells. The purity of the cell populations was verified directly after sorting as >98%. The viability of sorted cells exceeded 90% as assessed by trypan blue exclusion.

### RNA extraction and quantitative analysis using real-time RT-PCR

RNA extraction and real-time RT-PCR were performed as described previously [21], [22]. Glyceraldehyde-3-phosphate dehydrogenase (GAPDH) transcripts were measured as an internal control. Primer pairs used for each PCR reaction are as listed in the **Table 2**.

### Genomic DNA sequencing

Sequencing for *MED12* in myometrial and leiomyoma cells was performed by the Sanger DNA sequencing method. DNA from each sample was extracted using DNeasy blood and tissue kit (Qiagen, Germantown, MD). The desired DNA fragment was first amplified using AmpliTaqGold enzyme (Applied Biosystems, Foster City, CA). The PCR products were purified using QIAquick PCR Purification Kit (Qiagen) and the sequencing reactions were performed utilizing the Big Dye Terminator v.3.1 Kit (Applied Biosystems) according to the manufacturer's instructions. Sequencing was performed on an ABI3730 Automatic DNA Sequencer (Applied Biosystems) at the Northwestern University Genomics Core Facility. The sequence graphs were analyzed both manually and on a computer with the Sequence Scanner (Applied Biosystems) program.

### Cell Culture

Both LMSP and LMMP were cultured in DMEM/F12 1∶1 (GIBCO/BRL, Grand Island, NY) containing 10% FBS and grown in a humidified atmosphere with 5% CO_2_ at 37°C. For hormone treatment, cells were treated in the presence or absence of 17β-Estradiol (E_2_; 10^−7^ M; Sigma-Aldrich) and/or R5020 (P; 10^−7^ M; Perkin-Elmer, Boston, MA) in phenol red-free DMEM/F12 with 10% charcoal-stripped FBS for 7 days. An MTS assay using the Cell Titer 96 Aqueous One Solution Cell Proliferation Assay (Promega Corp., Madison, WI) was performed according to the manufacturer's instructions. The effects of the E_2_ and/or P on the viability of LMSP and LMMP were evaluated. Co-culturing in two separate compartments or with indirect contact with myometrial cells (MM) was performed using Transwell Permeable Supports (0.4-µm pore; Corning Incorporated, Pittston, PA). Side or main cell populations in mixed co-cultures with MM at a 1∶1 ratio were identified after initially dye-labeling each cell type. We labeled the cytosolic membranes of LMSP or LMMP cells using PKH-26 (red; Sigma-Aldrich), whereas myometrial cells were labeled by PKH-67 (green; Sigma-Aldrich). Cells originating from LMSP or LMMP in mixed co-cultures were identified using long-lasting status PKH-26.

### Immunofluorescence and histological analysis

Indirect immunofluorescence staining was performed described previously [11], [23]. Background fluorescence was determined by applying the secondary conjugated antibody alone and by replacement of the primary antibody with nonimmune serum. Slides were successively stained with various antibodies as listed in the **Table 1**, followed by incubation with secondary antibodies. Images were collected using an inverted Axiovert 200 fluorescent microscope (Carl Zeiss, Gottingen, Germany).

### Transplantation analysis

Northwestern University's Animal Care and Use Committee approved all procedures involving animals in this study. The protocol for the acquisition of surgical specimens was approved by Northwestern University's Institutional Review Board. LMSP or LMMP were co-cultured with primary myometrial cells for 4–6 days. The ratio of LMSP to myometrial cells before xenografting was 1∶9; and the ratio of LMMP to myometrial cells was 1∶7. Cells were collected from the culture plates by trypsin digestion and suspended into rat-tail collagen (type I) solution (BD Bioscience, San Jose, CA) at 10^5^ cells per 10 µl. We have successfully used this method to study the hormonal response of human endometrial tissue [24], [25]. With this technique, the low-density collagen gel consists mostly of water, and thus the pellet volume (10 µl) does not reflect the starting volume of the tumor. When cell pellets are incubated at 37°C overnight as floating cultures, they become smaller than 1 mm in diameter due to contraction of the collagen by the leiomyoma cells. Therefore, the estimated starting volume of the cell graft is smaller than 0.6 mm^3^. The grafting procedure was performed as described previously [25]. Cell pellets were grafted onto opposing kidneys of adult female non-obese diabetic-scid (IL2R*γ* ^null^) mouse hosts (Jackson Laboratory, Bar Harbor, ME). The cell pellets are high in water content, and thus, they become smaller under the pressure of the subrenal capsule. The estimated starting volume of tissue grafts under the renal capsule was approximately 1 mm^3^. According to our previous report, estrogen and progesterone are known to promote the growth of cells derived from uterine fibroids when transplanted under the kidney capsule [25]. For this reason, all hosts were ovariectomized and supplemented with sc implantation of 80 mg progesterone (P_4_; Sigma-Aldrich) plus 80 µg E_2_ (Sigma-Aldrich). These doses were chosen because previous studies demonstrated that they were able to sustain systemic E_2_ and P_4_ levels within cycling women [26]. The effects of ovariectomy and hormone treatments were confirmed by the gross appearance and histology of the host female reproductive tracts. The presence of hormone pellets was also confirmed at the time of termination of the host.

### Data analysis

Tumor volume was measured using the program ImageJ (National Institutes of Health, http://rsbweb.nih.gov.ezproxy.galter.northwestern.edu/ij/index.html). *P* value was calculated using the unpaired Student's t-test. *P* value less than 0.05 were considered statistically significant.

## Results

### Leiomyoma have a lower percentage of SP cells than normal myometrium

Since the SP phenomena has recently been shown to identify putative myometrial somatic stem cells in mice [5] and humans [9], [27], we evaluated differences in the SP between the myometrium and leiomyoma. Single cell suspensions of normal myometrium and leiomyoma tissue prepared from patients were each stained with Hoechst 33342 dye and analyzed by flow cytometry to identify the SP. PI staining was used to evaluate cell viability. In the representative experiment shown in Fig. 1A, there were nearly two-fold more SP cells in the normal myometrium of this patient compared to that found in the leiomyoma tissue. When reserpine, an inhibitor of ATP binding cassette transporter activity, was included as a control, no SP cells were detected, suggesting that the Hoechst dye efflux from the SP was due to ATP binding cassette transporter activity. Comparison of the two tissues from 10 patients showed that the normal myometrium had a consistently and significantly larger SP than leiomyomas (2.07%±0.46 vs. 1.19%±0.23, p<0.05, n = 10; Fig. 1B). Based on our recent finding that MMSP have a myometrial stem cell phenotype [9], we further characterized the SP of leiomyoma to identify leiomyoma stem cells.

### LMSP represent an immature or undifferentiated cell population

Real-time RT-PCR analysis of mRNA derived from isolated MMSP, MMMP, LMSP, and LMMP demonstrated that the expression levels of estrogen receptor-α (ESR1), progesterone receptor (PGR), and the smooth muscle cell markers, including αSMA, were present at very low levels in LMSP compared with both LMMP and whole leiomyoma tissues (Fig. 1C and D). These results suggest that LMSP represents an immature or undifferentiated cell population just after isolation, consistent with our observation that LMSP differentiate into uterine leiomyoma cells after co-culture with myometrial cells.

### LMSP express cell surface antigens different than those of blood cells, endothelial cells, or bone marrow-derived mesenchymal stem cells (MSC)

Phenotypic analyses using flow cytometry demonstrated that LMSP contain 5.6% CD31-positive cells and 2.3% CD45-positive cells. These results suggest that cell surface marker expression on LMSP differ from those on endothelial progenitor cells or hematopoietic stem cells. The percentage of CD31-positive cells in LMSP is lower compared with MMSP, whereas CD45 expression is rare in both LMSP and MMSP. We also analyzed the expression of the bone marrow MSC markers CD73, CD90, CD105 and Stro-1. In LMSP, expression levels of the markers were 23.3% for CD73, 27.1% for CD90, 2.3% for CD105, and 3.9% for stro-1 (Fig. 2A and B). Considering that these markers are commonly found on MSC, our data suggest that LMSP are not identical to MSC.

### Cell cycle analysis reveals that LMSP are quiescent

An important characteristic of hematopoietic and other tissue-specific stem cells is that they remain dormant or quiescent, arrested in the G_0_ phase of the cell cycle and protected from depletion or exhaustion [28], [29], [30]. Exit from G_0_ and entry into G_1_ are associated with an increase in transcription, which can be measured by staining with pyronine Y (PY), an RNA-specific dye. Co-staining with PY and Hoechst 33342, followed by flow cytometry analysis, revealed that 83.67%±5.73 of LMSP but only 58.50%±6.06 of LMMP were in the G_0_ phase (Fig. 2C), which is consistent with the existence of quiescent stem cells within LMSP.

### MED12 mutations demonstrate that LMSP are committed to a leiomyoma cell lineage

Recently, mutations in the *MED12* gene have been reported in the majority of leiomyoma tissues and cells [16]. We directly sequenced the *MED12* gene in 3 pairs of normal myometrium and adjacent leiomyoma tissues, as well as in main population and SP cells isolated from these samples. We found mutations involving a substitution of guanine (G) at the 131 bp position to either alanine (A) or cytosine (C) in leiomyoma tissue and LMSP and LMMP isolated from leiomyoma tissues, but not in adjacent myometrial tissues or their main population or SP cells (Fig. 3). Two patients had the same c.131G>A mutation shown in the upper panel of Fig. 3, and the third patient had the c.131G>C mutation shown in the lower panel. These two missense mutations affected the amino acid residue at codon 44, giving rise to conversions G44A and G44D (Fig. 3). These data suggest that LMSP carry a genetic mutation that may lead to tumorigenic transformation in cells already been committed to a leiomyoma cell lineage.

### Mixed co-culture with myometrial smooth muscle cells enhances initial attachment and spread and eventual differentiation and growth of LMSP

To further characterize LMSP *in vitro*, we attempted to culture and expand these cells using conventional media supplemented with E_2_ and/or P. LMSP cells cultured alone never attached to plastic or spread up to 4 weeks in culture (Fig. 4A). LMSP also failed to grow in a co-culture system in which Transwell Permeable Supports were used to create separate compartments of confluent myometrial cells and seeded LMSP (Fig. 4B). Finally, we tried a mixed co-culture system, in which equal numbers (50,000) of LSMP and myometrial smooth muscle cells were mixed 1∶1 and maintained in the same culture dish. In mixed co-culture, the LMSP attached and spread together with unsorted myometrial cells *in vitro* in DMEM/F12 1∶1 10% FBS without any hormonal treatment (Fig. 4C), reaching confluence in a 6-cm culture dish within 2 weeks. In this co-culturing system, we labeled LMSP or LMMP using PKH-26 fluorescent dye. No fusion between the two cell types was observed. After mixed co-culture, the final ratio of LMSP to LMMP was approximately 1∶9. This result indicates that LMSP did attach and spread but did not increase significantly in number during the first mixed co-culture. These findings suggest that cell-cell interactions and/or myometrial cell-derived secretory factors may be required for attachment and survival of LMSP.

### LMSP differentiate to LMMP and proliferate after co-culture

After grown to confluence in the first mixed co-culture plate with myometrial cells, LMSP cells were sorted by flow cytometry using a PKH-26 dye, and their gene expression profile was determined using real-time PCR. The steroid hormone receptors, ESR1 and PGR, and smooth muscle cell markers were expressed at the same level in LMSP, LMMP, and cultured cells from whole leiomyoma tissue (Fig. 4D and E). Immunostaining showed that protein expression of αSMA was not different between LMSP and LMMP after these cells were maintained in mixed co-culture (Fig. 4F). Because LMSP did not express these markers before mixed co-culture with myometrial smooth muscle cells, these results indicate that LMSP differentiate to a cell type similar to LMMP during co-culture. Moreover, after sorting and re-culturing, the pure LMSP population grew to full confluence indicating that the differentiated LMSP population now gained the potential for proliferation similar to that observed in LMMP (Fig. 4F). αSMA-positive cell frequency during this second culture was 100% (Fig. 4F). FACS analysis was performed to demonstrate the purity of LMSP and LMMP (control) cell types.

### Generation of human leiomyoma-like tumors from LMSP in immunodeficient mice

Single-cell suspensions of 100,000 cells obtained from mixed co-culture systems were transplanted under the kidney capsule of ovariectomized immunosuppressed mice treated with E_2_ and P_4_ for 12 weeks (see Materials and Methods). Each cell pellet from a co-culture system included myometrial smooth muscle cells (MM) plus LMMP or MM plus LMSP. Mice inoculated with a single cell suspension (100,000 cells) cultured from whole myometrium (MM only) were used as negative controls, as these cells never grow into tumors [25]. To assess the capacity for tumorigenesis, each xenografted cell pellet was analyzed for (i) tumor formation, (ii) the 3 dimensions and volume of the tumor, (iii) protein expression using immunohistochemistry (Fig. 5). We demonstrated that both LMSP+MM and LMMP+MM were capable of generating human leiomyoma tumors; however the tumor size derived from LMSP+MM was significantly larger (3.67 mm^3^±1.07) compared with LMMP+MM-derived tumors (0.54 mm^3^±0.20, p<0.05, n = 10 paired patient samples; Fig. 5A and B). The tumors were characterized by the presence of αSMA-positive cells in the renal capsule (Fig. 5C). We confirmed the expression of estrogen receptor (ER) and progesterone receptor (PR) in all the xenografts obtained from animals grafted with LMSP+MM. Consistent with the formation of larger tumors, LMSP+MM-derived tumor cells displayed significantly higher proliferation as determined by a higher Ki67 labeling index (2.38%±0.12) compared with LMMP+MM-derived tumors (0.35%±0.08) (Fig. 5C and D).

## Discussion

Uterine leiomyomas are monoclonal tumors, with growth of the neoplasm occurring via clonal expansion from a single cell; this raises the possibility for development of new, targeted therapeutic interventions [31]. Our results reinforce the hypothesis that LMSP are likely involved in leiomyoma tumorigenesis. Most leiomyomas contain specific genetic mutations suggesting that transformation of normal myocytes into abnormal myocytes is required at some point during the genesis of a leiomyoma [16]. This process appears to be quite common, in view of the high prevalence of microscopic leiomyomas. We believe that LMSP arise from this myometrial transformation, though the timing of this transformation has not been elucidated and may occur in adults or during an embryonic stage.

The myometrium itself has a prominent regenerative capacity. Marked pregnancy-induced expansion of the human uterus, mainly composed of myometrial cells, is repeated multiple times throughout the reproductive life of an individual. As uterine leiomyomas are thought to be derivatives of myometrial cells [13], [14], we hypothesize that a population of putative stem/reservoir cells that support growth exists within the leiomyoma. In support of this hypothesis, the Hoechst-stained cells contained a small fraction of SP cells that were resting in G_0_ phase, a characteristic of stem cells that allows them to persist in various tissues. Real-time RT-PCR data revealed that the LMSP rarely expressed steroid hormone receptors and smooth muscle cell markers. However, after culture, these markers were expressed naturally at the same levels as seen in LMMP and the total leiomyoma fraction. These results indicate that LMSP represent a population of cells that exist in an undifferentiated state within the leiomyoma that have the potential to differentiate into uterine leiomyoma cells within the environment of the uterine myometrium.

Steroid hormones play a role in the growth of uterine leiomyomas and endometrium [4], [32], [33], [34], [35]. Upregulation of ESR1, PGR and aromatase in leiomyoma tissue were reported [36]. The enzyme aromatase, which is encoded by the CYP19A1 gene produces estrogens, appears to be an important regulator of estrogen response in leiomyomas. Progesterone, through its receptor, PGR also increase leiomyoma growth via inhibiting apoptosis and promoting cell proliferation [37], [38], [39].

To follow our previous work, we continued to employ Hoechst 33342 based isolation of SP cells to study stem/reservoir cell biology in uterine leiomyoma [9]. It would desirable to use an independent methodology such as cell sorting via a surface antigen for the isolation of a leiomyoma cell population enriched in stem cells. Unfortunately, such a marker to be used for this purpose has not been published to date.

Intriguingly, we found that LMSP have tumorigenic capacity under E_2_+P_4_ stimulation, despite our finding that LMSP are negative for ESR1 and PGR. In mammary structures of humans and mice, mammary stem cells, despite being void of ESR1 and PGR themselves, are subject to regulation in both number and repopulating ability by steroid hormones, particularly progesterone [40], [41], [42], [43]. We hypothesize that paracrine factors mediate signals from steroid receptor-positive adjacent to LMSP. The most important characteristic of stem cells is their ability to generate tumors in immunocompromised mice. Tumors originating from LMSP grew larger than those originating from LMMP, confirming the capacity of leiomyomas to initiate and sustain tumor growth.

Thorough characterization of LMSP is necessary to understand the complex mechanisms underlying the pathogenesis of leiomyoma, and our procedure for isolating and cultivating LMSP have made such studies possible. The elucidation of the functions and cellular properties of LMSP will broaden our understanding of leiomyoma pathophysiology and will be the focus of future studies.
